# Cultured brain pericytes adopt an immature phenotype and require endothelial cells for expression of canonical markers and ECM genes

**DOI:** 10.3389/fncel.2023.1165887

**Published:** 2023-05-02

**Authors:** Fabiana Oliveira, Olga Bondareva, Jesús Rafael Rodríguez-Aguilera, Bilal N. Sheikh

**Affiliations:** ^1^Helmholtz Institute for Metabolic, Obesity and Vascular Research (HI-MAG) of the Helmholtz Center Munich, Leipzig, Germany; ^2^Medical Faculty, Leipzig University, Leipzig, Germany

**Keywords:** brain, vasculature, pericytes, single cell RNA-seq, endothelial, extracellular matrix

## Abstract

Pericytes (PCs) are essential components of the blood brain barrier. Brain PCs are critical for dynamically regulating blood flow, for maintaining vascular integrity and their dysregulation is associated with a myriad of disorders such as Alzheimer’s disease. To understand their physiological and molecular functions, studies have increasingly focused on primary brain PC isolation and culture. Multiple methods for PC culture have been developed over the years, however, it is still unclear how primary PCs compare to their *in vivo* counterparts. To address this question, we compared cultured brain PCs at passage 5 and 20 to adult and embryonic brain PCs directly isolated from mouse brains via single cell RNA-seq. Cultured PCs were highly homogeneous, and were most similar to embryonic PCs, while displaying a significantly different transcriptional profile to adult brain PCs. Cultured PCs downregulated canonical PC markers and extracellular matrix (ECM) genes. Importantly, expression of PC markers and ECM genes could be improved by co-culture with brain endothelial cells, showing the importance of the endothelium in maintaining PC identity and function. Taken together, these results highlight key transcriptional differences between cultured and *in vivo* PCs which should be considered when performing *in vitro* experiments with brain PCs.

## 1. Introduction

Pericytes (PCs) are mural cells that are embedded in the basement membrane of microvessels (Armulik et al., 2011; Winkler et al., 2011). Together with endothelial cells (ECs), PCs play a major role in vascular function through the regulation of angiogenesis (Ozerdem and Stallcup, 2003; Chang et al., 2013), vessel integrity (Bjarnegaård et al., 2004; Armulik et al., 2010; Bell et al., 2010), and extracellular matrix composition (Stratman et al., 2009; Sava et al., 2015). The interaction between PCs and ECs is first established during embryogenesis, where mesenchymal cells originating in the mesothelium or the neural crest (Etchevers et al., 2001; Korn et al., 2002) are recruited to the microvessels by EC-secreted Platelet-derived growth factor subunit B (PDGF-B) (Enge et al., 2002; Bjarnegaård et al., 2004). Transforming growth factor beta (TGF-β) signaling is then involved in mediating the proliferation and differentiation of PCs during vessel development (Winkler et al., 2011). In the mature microvasculature, the endothelial-to-pericyte ratio is varied across different organs, but it is the highest in the central nervous system (Armulik et al., 2011). There, PCs maintain the blood brain barrier (BBB) and are responsible for its maturation and stability (Armulik et al., 2011). Interestingly, PC deficiency in the CNS has been linked to multiple diseases, including Alzheimer’s disease, diabetic retinopathy and neonatal intraventricular hemorrhage (Ballabh, 2010; Hammes et al., 2011; Zlokovic, 2011). Furthermore, animal models with low PC density show an increase in BBB breakdown and premature onset of neurodegenerative disorders (Bjarnegaård et al., 2004; Bell et al., 2010). Thus, further studies of PC biology could improve our understanding of their role in vascular homeostasis and how they relate to disease.

Multiple *in vitro* models of the BBB have been developed over the years with the goal of reliably reproducing the physiological conditions of microvasculature in a straight-forward and accessible manner. As a key component of the vessels, PCs are often included in BBB models as primary cells (Prashanth et al., 2021), which can be isolated from the mouse brain (Dore-Duffy, 2003; Tigges et al., 2012). Protocols for PC isolation typically involve an initial digestion of the brain into single cell suspensions or microvessel fragments, followed by the selective subculture of primary PCs (Balabanov et al., 1996; Nees et al., 2012; Boroujerdi et al., 2014; Thomsen et al., 2015; Smyth et al., 2018a). When cultured in an appropriate medium, primary mouse brain PCs are thought to consistently express PC markers (PDGFRB, NG2, and CD146) and can remain proliferative for 40 passages (Tigges et al., 2012). However, multiple reports have suggested that cultured PCs show stem cell potential and can be easily differentiated into other cell types *in vitro*, including mesenchymal stem cells, myogenic cells, osteocytes, chondrocytes and adipocytes (Farrington-Rock et al., 2004; Dore-Duffy et al., 2006; Crisan et al., 2008). Therefore, it is still unclear how primary mouse brain PCs compare to *in vivo* microvascular PCs and if they remain pure after prolonged culture.

In this study, we used single-cell RNA sequencing to compare cultured PCs with *in vivo* brain PCs. We observed that early and late passage PCs display low heterogeneity and are most similar to embryonic PCs. Our analysis also indicated that mono-cultured PCs express lower levels of canonical PC marker and extracellular matrix (ECM) genes, especially laminins and integrins. However, when co-cultured with ECs, PCs upregulate their canonical markers and ECM-related genes, indicating that the contact with other ECs is essential for the maintenance of PC identity and function.

## 2. Materials and methods

### 2.1. Animals

C57BL/6N mice were housed at 22 ± 2°C and maintained in a 12 h day, 12 h night cycle. Water and food were provided *ad libitum*. All experiments were performed in accordance with the animal ethics laws of Saxony, Germany, and were approved by the state animal ethics committee (Landesdirektion Sachsen, Leipzig, Germany).

### 2.2. Primary PC isolation and culture

Two sets of PCs were isolated as previously described, with minor modifications (Tigges et al., 2012; Sheikh et al., 2020). Briefly, for each primary PC isolation, brains of 6 female wild type mice (6–8 weeks old) were dissected and placed in cold minimum essential media (MEM) (ThermoFisher, 41090028). The olfactory bulb, cerebellum and medulla were removed, and the brain was thoroughly minced with a scalpel. After a wash with MEM and centrifugation at 300 × *g* for 5 min, the tissue was incubated with 3 ml of enzymatic solution for 70 min at 37°C while being mixed every 10 min with tube inversions. The enzymatic solution was prepared using the Papain dissociation system (Worthington, LK003150) with a final concentration of 30 U/ml papain (containing 1.6 mM L-cysteine and 0.8 mM EDTA) and 40 μg/ml DNase I in Earl’s balanced salt solution. The digested brain was then passed 10 times through an 18 gauge needle and subsequently 10 times through a 21 gauge needle. Following a wash with MEM and centrifugation at 300 × *g* for 5 min, 3 ml of the digested brain solution was transferred to a 15 ml Falcon tube and layered on top of 5 ml of 22% BSA (Cytiva, SH30574.02) in PBS. In order to separate the myelin, the solution was centrifuged at 1000 × *g* for 10 min and the top lipid layer was carefully removed. The cell pellet was resuspended in 5 ml of endothelial cell growth media [ECGM: 10% FCS, 25 μg/ml heparin (Sigma, H3149-10KU), 1 μg/ml Ascorbic acid (Sigma, A4403-100 mg), 2 mM GlutaMax (Thermo Fisher, 35050061), 1 × Pen/Strep (Thermo Fisher, 15140122) and 30 μg/ml ECGS (Millipore, 02–102) in Hams F12 (ThermoFisher, 21765029)] and centrifuged for 5 min at 300 × *g*. Two wells of a 6-well plate were pre-coated with 0.02% collagen type I in sterile water for at least 2 h followed by two gentle washes with sterile water and one wash with PBS. The isolated cell pellet was resuspended in ECGM and cultured in the collagen coated plate. After 20 h of culture, the cells were washed 3 times with PBS, and fresh ECGM was added. The medium was replenished every 3 days and the cells were kept in culture until they reached confluency (∼9 days). The PCs were passaged (1:4) to fresh collagen coated 6-well plates for five passages until they were ready for use. ECGM was used in the first two passages and Pericyte Medium (1% Penicillin/Streptomycin, 2% fetal bovine serum, and 1% Pericytes Growing Supplements) (ScienCell, 1201) from passage 2 onward. PC identity was confirmed via PDGFRβ expression.

### 2.3. FACS isolation of brain pericytes

Brains were dissected and rinsed in ice-cold PBS. The olfactory bulb and cerebellum were removed. The brain was then dissociated with the Neural Dissociation kit P (Miltenyi Biotec, 130-092-628), as per the manufacturer’s instructions, via the gentleMACS Octo Dissociator system with heaters (MACS Technology, Miltenyi Biotec) using the 37C_NTDK_1 program. Cells were transferred via a 20-gauge syringe through a 70 μm cell strainer and into a 50 ml Falcon tube. Cells were collected by centrifugation (4°C, 300 × *g*, 5 min). Myelin was removed using myelin removal beads (Miltenyi Biotec, 130-096-733), as per the manufacturer’s instructions, before staining. FACS isolation of PCs was performed using the EMBRACE technology (Sheikh et al., 2019, 2020). Briefly, samples were stained with PDGFRβ (R&D Systems, AF1042: 1:50), CD45-PE (BD Pharmingen, 533081; 1:300), CD31-APC (eBioscience, 17-0311-85; 1:250), and CD102-BV421 (BD Pharmingen, 740018; 1:250) antibodies diluted in FACS buffer (2% fetal calf serum in PBS). The PDGFRβ was conjugated to CF488 (FITC channel) using the CF488A antibody labeling kit (Sigma) as per the manufacturer’s instructions. Staining was done on ice in a total volume of 300 μl. Cells were washed in 14 ml of FACS buffer, collected by centrifugation (4°C, 300 × *g*, 5 min), resuspended in FACS buffer containing 1 μg/ml propidium iodide and passed through a 100 μm cell strainer into a FACS tube. Cells were sorted on the FACS Aria instrument (BD Biosciences). Single cells were selected based on forward and side scatter. Dead cells were removed using propidium iodide. Pericytes were gated based on PDGFRβ*^high^*, CD31^–^, CD45^–^, and CD102^–^ expression.

### 2.4. Single cell workflow

One set of PCs isolated from 6 mice was used for the P5 timepoint, while a different, second set of PCs were used for the P20 timepoint. Single cell RNA-seq was performed using 10 × Next GEM Single Cell 3′ GEM kit v3.1 (10 × Genomics; Pleasanton, CA, USA) according to the manufacturer’s protocol. Briefly, passage 5 PCs and passage 20 PCs as well as freshly sorted brain PCs were spun down (4°C, 300 g, for 5 min), resuspended at ∼1000 cells/μl, and immediately loaded into the 10 × Chromium controller. Generated libraries were sequenced on an Illumina NovaSeq with > 45.6 × 10^3^ reads per cell followed by de-multiplexing and mapping to the mouse genome (build mm10) using CellRanger v5.0 (10 × Genomics).

### 2.5. Bioinformatical analysis

Our established workflow was used to analyze the data (Bondareva et al., 2022). Briefly, gene expression matrices of passage 5 PCs, passage 20 PCs and brain PCs were obtained using the CellRanger software v5.0.1 (10 × Genomics). Additionally, two different datasets were included in the analysis: adult mouse brain vascular cells (GSE98816) (Vanlandewijck et al., 2018) and embryonic mouse brain PCs (GSE133079) (Sheikh et al., 2019). The next steps were performed with the Seurat package v3.0 (Satija et al., 2015). Initially, data were filtered for cells with more than 1000 uniquely expressed genes, and with less than 20% of overall reads mapping to the mitochondrial genome. Next, we merged the data with merge() function, normalized it with NormalizeData() and identified 2000 highly variable genes with the function FindVariableFeatures(). Then, the data was scaled with ScaleData() and the variables linked to the percentage of mitochondrial and ribosomal genes were regressed. After using the command RunPCA(), the data was clustered using the first 15 dimensions at a 0.8 resolution with the functions FindNeighbors(), FindClusters() and RunUMAP(). Clusters were labeled according to cell markers for PCs (*Pdgfrb*, *Cspg4*, *Anpep*), smooth muscle cells (*Acta2*, *Myh11*, *Tagln*), endothelial cells (*Pecam1*) microglia (*Csf1r*, *Cd48*, *Emr1*), fibroblasts (*Pdgfra*, *Lum*, *Dcn*, *Col3a1*), oligodendrocytes (*Mobp*, *Mag*, *Plp1*) and astrocytes (*Aldh1l1*, *Slc4a4*). The clusters that remained unidentified were labeled according to their original datasets: PCs passage 5 (PC5), PCs passage 20 (PC20) and embryonic brain PCs (embPC). Differentially expressed genes were found with the function FindMarkers(): upregulated genes were assigned as > 1.0 log_2_ fold change (log_2_FC), conserved genes as −0.5 < log_2_FC < 0.5 and downregulated genes as −0.5 < log_2_FC. Finally, we used the corrplot package v0.92 (Friendly, 2002) for the analysis of correlation between clusters. The average gene expression of each cluster was calculated with the command AverageExpression(), the Pearson correlation was obtained with cor() and a correlation matrix was made with the corrplot.mixed() function.

### 2.6. PC co-culture and FACS sorting

Primary mouse PCs at passage 8 were stained with Vybrant™ CFDA SE Cell Tracer Kit (Invitrogen, V12883) as specified by the manufacturer. Briefly, PCs were treated with CFDA SE (50 μM) in PBS for 15 min in the incubator. Then, the solution was replaced with Pericyte Medium (ScienCell, 1201) and the cells were incubated for an additional 30 min. The stained passage 8 primary mouse PCs were dissociated and mixed with untreated mouse brain bEnd.3 endothelial cells (Montesano et al., 1990) in a proportion of 1:10. The cells were then seeded into a coated plate (0.02% collagen type I) and co-cultured for 3 days. As a control, CFDA SE-dyed passage 8 primary mouse PCs were cultured in a monoculture for 3 days without ECs. The media used was 50% Pericyte Medium and 50% bEnd.3 medium. bEnd.3 medium consisted of 10% FCS, 1% Glutamax (ThermoFisher, 35050061) and 1% Pen/Strep (Thermo Fisher, 15140122) in dulbecco’s modified eagle’s medium (DMEM) (Gibco, 41966-029).

After 3 days, the co-cultured cells were trypsinised and stained with 5 μl Zombie NIR™ dye (BioLegend, 77184) in 500 μl of PBS and incubated for 15 min in the dark. Then, the suspension was washed with 10 ml of PBS and resuspended in FACS buffer (2% FCS in PBS). The cells were sorted on the FACS Melody instrument (BD Biosciences). Side and forward scatter data was used to identify single cells. Dead cells were removed based on the Zombie dye. PCs were gated and sorted using the CFDA SE staining.

### 2.7. RNA isolation, cDNA synthesis, and qPCR

Pericytes RNA was isolated with the RNeasy Mini Kit (Qiagen, 74106). The cDNA was synthesized with Maxima first-strand cDNA synthesis kit (Thermo Fisher Scientific, K1671). Finally, qPCR reactions were run with Power SYBR Green PCR master mix (Life Technologies, 4368708) on an LC480 instrument (Roche). The resulting Cp values were standardized according to a standard curve, converted from a log to a linear scale, and the expression data were normalized to *Gapdh* values. Details of the primers sequences are provided in Supplementary Table 1.

### 2.8. Immunostaining

Passage 9 PCs were cultured for 3 days in 8 well μ-slides (Ibidi, 80826). Cells were fixed with 4% formaldehyde for 10 min and then washed 3 times with PBS. The slides were blocked for 2 h with 1% basic serum albumin (PanReac AppliChem, A1391) and 0.1% Triton X-100 (Roth, 3051.3) in PBS, and then incubated overnight with PDGFRβ antibody (1:100, R&D Systems, AF1042). Cells were washed 3 times with blocking solution and incubated with an AlexaFluor 488 donkey anti-goat IgG antibody (1:400, Thermo Fisher Scientific, A11055). Pictures were obtained with a confocal microscope (Zeiss, LSM980) and the same exposure and acquisition settings were used for all cells.

### 2.9. Statistics

Normalized gene expression data after standardization to *Gapdh* were analyzed using GraphPad Prism v8.0. Data are presented as mean ± SEM Data were analyzed using a two-tailed Student’s *t*-test.

## 3. Results

### 3.1. Cultured brain PCs are homogenous and downregulate canonical markers

**FIGURE 1:**
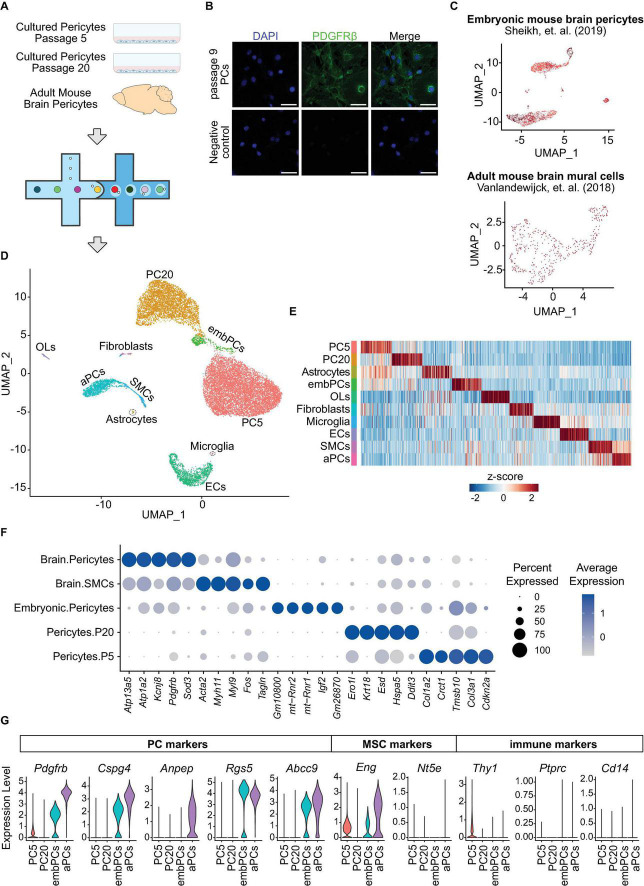
Cultured PCs are a homogenous population and downregulate their canonical markers. **(A)** Experimental design: scRNA-seq analysis of adult mouse brain PCs and cultured primary mouse PCs at passage 5–20 was undertaken via the 10x genomics platform. **(B)** Immunostaining of PDGFRβ in primary brain PCs at passage 9. Scale bars, 50 μm. **(C)** Uniform manifold approximation and projection (UMAP) clustering of the two additional datasets included in the study: embryonic mouse brain PCs (GSE133079) (Sheikh et al., 2019) and adult mouse brain mural cells (GSE98816) (Vanlandewijck et al., 2018). **(D)** UMAP clustering of all cells included in the study. Colors and labeling indicate the cell types. **(E)** Heatmap showing the 100 most enriched genes per cluster. Colors represent the average z-score of a cluster for each gene. **(F)** Dot plot indicating the average expression of the top 5 most highly enriched genes in PC and SMC clusters. **(G)** Violin Plot representing the expression of 5 PC markers (*Pdgfrb*, *Cspg4*, *Anpep*, *Rgs5*, and *Abcc9*), mesenchymal stem cell markers *Eng* and *Nt5e*, as well as the immune cell markers *Thy1*, *Ptprc* and *Cd14* in PC5, PC20, aPCs, and embPCs. aPCs, adult brain pericytes. embPCs, embryonic brain pericytes. ECs, endothelial cells. OLs, oligodendrocytes. PCs, pericytes. PC5, passage 5 pericytes. PC20, passage 20 pericytes. SMCs, smooth muscle cells.

Single cell (sc)RNA-seq is a powerful technique for identifying heterogeneity and phenotypic shifts in vascular cells (Bondareva and Sheikh, 2020; Kalucka et al., 2020). To uncover if cultured PCs are transcriptionally similar to *in vivo* PCs, we performed scRNA-seq analysis of two independent cultures of primary mouse brain PCs, one at passage 5 and the other at passage 20, as well as adult mouse brain PCs (Figures 1A, B and Supplementary Figure 1A). Additionally, two further datasets were included in the study: adult mouse brain vascular cells (GSE98816) (Vanlandewijck et al., 2018) and embryonic mouse brain PCs (GSE133079) (Sheikh et al., 2019; Figure 1C). Cells with low feature counts (<1000 unique genes) or high percentage of mitochondrial genes (>20% of transcripts) were filtered out (Supplementary Figure 1B) leaving a total of 17,630 cells to be analyzed in the study. Unbiased clustering identified 18 clusters (Supplementary Figure 1C) that were later combined into 10 according to their similarities (Figures 1D, E and Supplementary Figures 1D–F). All clusters displayed high expression of the female-specific gene *Xist*, indicating that the majority of cells in the datasets originated from female mice (Supplementary Figure 1G). The labeling of each cluster was based on canonical cell type markers (Figure 1F and Supplementary Figure 1H; Vanlandewijck et al., 2018). Three clusters had unclear markers and were labeled according to their origin (Supplementary Figure 1E). Four distinct clusters were categorized as PCs: adult brain PCs (aPCs), embryonic brain PCs (embPCs), passage 5 PCs (PC5) and passage 20 PCs (PC20).

We checked the expression of canonical PC markers in each PC cluster (*Pdgfrb*, *Cspg4*, *Anpep*, *Rgs5*, and *Abcc9*). aPCs displayed consistently high expression of canonical PC markers compared to other clusters (Figure 1G). EmbPCs had intermediate expression of canonical PC markers, while cultured PCs showed the lowest expression. All four PC clusters displayed low levels of smooth muscle cell markers (Figure 1F). As cultured PCs are thought to possess stem cell activity *in vitro* (Dore-Duffy et al., 2006), we also investigated if cultured PCs expressed markers for mesenchymal stem cells (MSCs). The expression of MSC markers was generally low in all four PC clusters (Figure 1G and Supplementary Figure 1H). Similarly, the expression of immune cell markers such as *Ptprc* was not detectable (Figure 1G).

**FIGURE 2:**
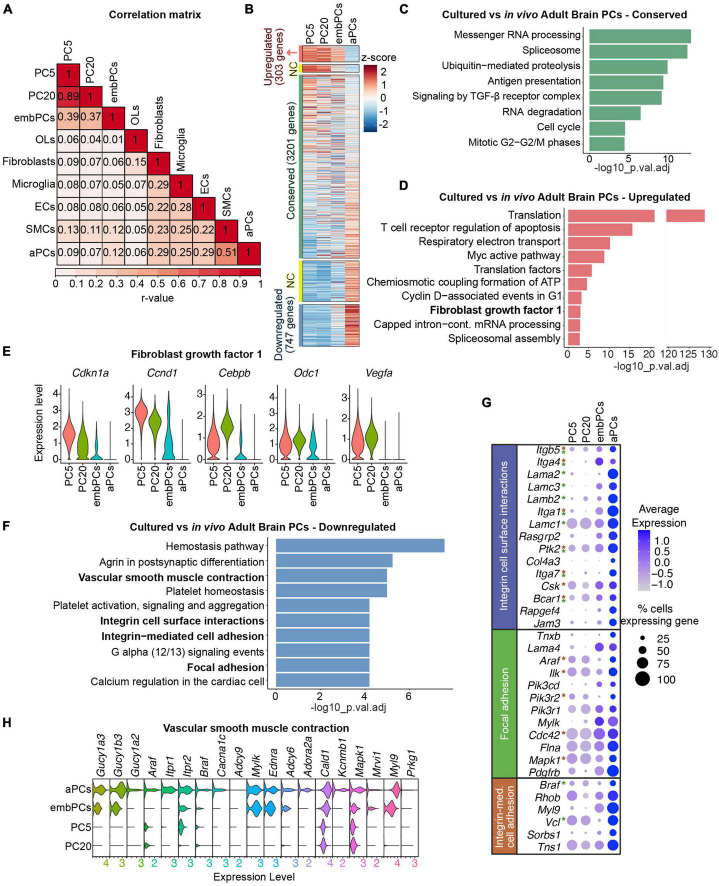
Cultured PCs deregulate ECM genes. **(A)** Correlation of gene expression between the 9 main clusters. Numbers represent Pearson’s correlation (*r*-value) **(B)** Heatmap representing the enrichment of gene expression in cultured, embryonic and adult PCs. Colors represent the average z-score for each gene. NC indicates genes not considered in the analysis, either 0.5 < log_2_FC < 1 or–1 < log_2_FC < –0.5. **(C)** BioPlanet annotated pathways conserved in cultured PCs vs. aPCs. **(D)** BioPlanet annotated pathways significantly upregulated in cultured PCs vs. aPCs. The highlighted FGF1 pathway is shown in more detail in **(E)**. **(E)** Violin plot representing the expression level of genes in the fibroblast growth factor 1 pathway in PC5, PC20, embPCs, and aPCs. **(F)** BioPlanet annotated pathways significantly downregulated in cultured PCs vs. aPCs. Pathways in bold are shown in more detail in **(G,H)**. **(G)** Dot plot representing the average expression of selected genes belonging to downregulated pathways in cultured PCs. Genes with asterisks are also part of other downregulated pathways, indicated by the colors (i.e., green for the focal adhesion pathway, and brown for the integrin-mediated cell adhesion). **(H)** Violin plot representing the expression level of genes in the vascular smooth muscle contraction pathway in PC5, PC20, embPCs, and aPCs. ECs, endothelial cells; NC, not considered; OLs, oligodendrocytes; PCs, pericytes; PC5, passage 5 pericytes; PC20, passage 20 pericytes; SMCs, smooth muscle cells.

Single cell studies have shown a continuum between pericytes and smooth muscle cells (Vanlandewijck et al., 2018; Muhl et al., 2020). Thus, we investigated whether cultured PCs showed any heterogeneity in their profiles. We observed a single cluster for PC5 and PC20 cells, indicating that the cultured cells are a homogeneous population (Supplementary Figures 1C–F). Indeed, even when searching for subclusters, we observed a strong overlap in transcriptional profiles of cultured PCs (*r* > 0.9, Supplementary Figures 1C, D). Furthermore, passage 5 and 20 PCs also displayed a strong overlap in their transcriptional profiles (*r* = 0.89, Figure 2A), suggesting that cultured PCs retain a similar state despite culture length. Altogether, our initial analysis indicates that cultured PCs are a homogeneous population that do not express stem cell markers, but do downregulate the expression of canonical PC markers.

### 3.2. Integrins and laminins are downregulated in cultured PCs

To understand how similar cultured PCs are to their *in vivo* counterparts, we compared the expression profiles of the 4 identified PC clusters. Interestingly, both PC5 and PC20 displayed a higher correlation score to embPCs (*r* = 0.39) than to aPCs (*r* < 0.1, Figure 2A). While embPCs indeed displayed the highest similarity to cultured PCs, aPCs showed the strongest correlation to adult smooth muscle cells (SMCs, *r* = 0.51), which is consistent with the continuum between PCs and SMCs in the adult brain (Vanlandewijck et al., 2018; Muhl et al., 2020).

We initially compared the expression profiles of PC5 and PC20 to aPCs. Out of the 5129 genes expressed in all 3 groups, 303 were upregulated (log_2_FC > 1), 3201 were conserved (−0.5 < log_2_FC < 0.5) and 747 were downregulated (log_2_FC < −1) in cultured PCs (Figure 2B). In this comparison, all 3 clusters had conserved pathways linked to housekeeping functions such as messenger RNA processing (Figure 2C), suggesting that essential cellular functions are not affected *in vitro*. However, pathways associated with translation, respiratory electron transport and ATP biosynthesis were upregulated in PC5 and PC20 (Figure 2D), suggesting a more metabolically active state *in vitro*. Additionally, PC5 and PC20 displayed an upregulation of fibroblast growth factor I genes (*Cdkn1a*, *Cebpb*, *Ccnd1*, *Odc1*, *Vegfa*) (Figures 2D, E). Differential expression (DE) analysis revealed the downregulation of genes associated with cell surface interaction and adhesion in PC5 and PC20 (Figure 2F), including integrins (*Itga1*, *Itga4*, *Itga7*, *Itgb5*) and laminins (*Lama2*, *Lama4*, *Lamb2*, *Lamc3*) (Figures 2F, G). Pathways related to vascular smooth muscle cell contraction were also downregulated (Figures 2F, H), suggesting some functional characteristics of mature brain PCs are lost *in vitro* (Nortley et al., 2019). Taken together, these results indicate that while a high number of genes are conserved between PC5, PC20, and aPCs, cultured PCs also display a striking downregulation of genes associated with PC maturity and differentiation, especially ECM genes (Figures 2F–H and Supplementary Figure 2).

### 3.3. Cultured PCs share similarities with embryonic brain PCs

**FIGURE 3:**
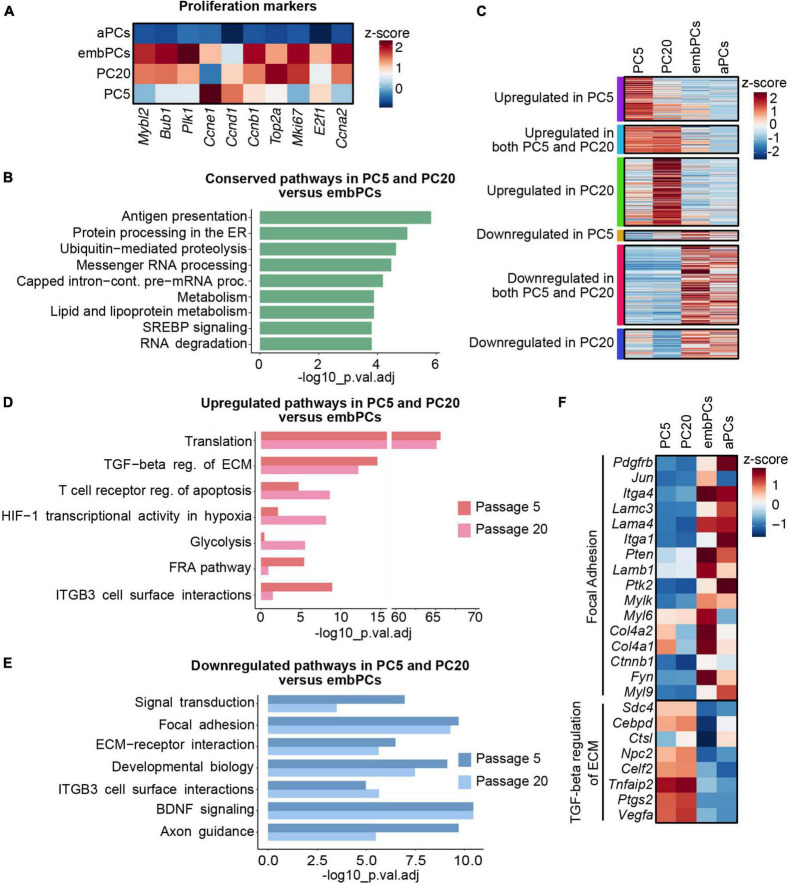
Extracellular matrix (ECM) and cell surface proteins are deregulated in cultured PCs compared to embPCs. **(A)** Heatmap depicting the enrichment of cell proliferation markers in PC5, PC20, aPCs, and embPCs. Colors represent the average z-score of a cluster for the indicated gene. **(B)** BioPlanet annotated pathways conserved in cultured PCs and embPCs. **(C)** Heatmap of selected genes significantly enriched in PC5, PC20, embPCs, and aPCs. Colored boxes on the left indicate if the genes represented in the plot are significantly deregulated in one or more of the cell clusters. **(D)** BioPlanet annotated pathways significantly upregulated in PC5 and/or PC20 in comparison to embPCs. **(E)** BioPlanet annotated pathways significantly downregulated in PC5 and PC20 in comparison to embPCs. **(F)** Heatmap depicting the enrichment of “focal adhesion” and “TGF-beta regulation of ECM” genes across PC5, PC20, aPCs, and embPCs. Colors represent the average z-score of a cluster for the indicated gene. aPCs, adult brain pericytes; embPCs, embryonic brain pericytes; PCs, pericytes; PC5, passage 5 pericytes; PC20, passage 20 pericytes.

In our analysis, embPCs and cultured PCs had a high correlation score (*r* = 0.39 with PC5 and *r* = 0.37 with PC20, Figure 2A). This similarity might be a reflection of the highly proliferative state of PCs during CNS angiogenesis and *in vitro* (Armulik et al., 2011). Indeed, the expression of proliferation markers in embPCs, PC5 and PC20 was significantly higher than in aPCs (Figure 3A). We then explored the similarities between embryonic and cultured PCs. A DE analysis indicated that a total of 5603 genes are conserved in PC5 and 6708 genes in PC20 when compared to embPCs. Additionally, pathway enrichment analysis showed that pathways related to RNA and protein processing as well as lipid and fatty acid metabolism are conserved in PC5, PC20, and embPCs (Figure 3B). These data suggests that embryonic and cultured PCs have a comparable protein and metabolic demand, possibly associated with their higher proliferative state.

Despite their similarities, multiple genes were upregulated in cultured PCs when compared to embPCs (Figure 3C). The enrichment analysis showed an upregulation of pathways associated with translation and TGF-β regulation of ECM in cultured PCs (Figure 3D). Alternatively, the focal adhesion and the ECM-receptor interaction pathways were downregulated in cultured PCs, in particular integrins (*Itga1*, *Itga4*), laminins (*Lama4*, *Lamc3*), and collagens (*Col4a1*, *Col4a2*) (Figures 3E, F). Notably, some of the downregulated ECM genes had high expression levels in aPCs (*Itga1*, *Itga4*, *Lama4*, *Lamc3*) (Figure 3F). These results indicate that PC5 and PC20 have a strong deregulation of ECM-related genes when compared with both adult and embryonic brain PCs.

### 3.4. ECM genes are deregulated in prolonged culturing of PCs

**FIGURE 4:**
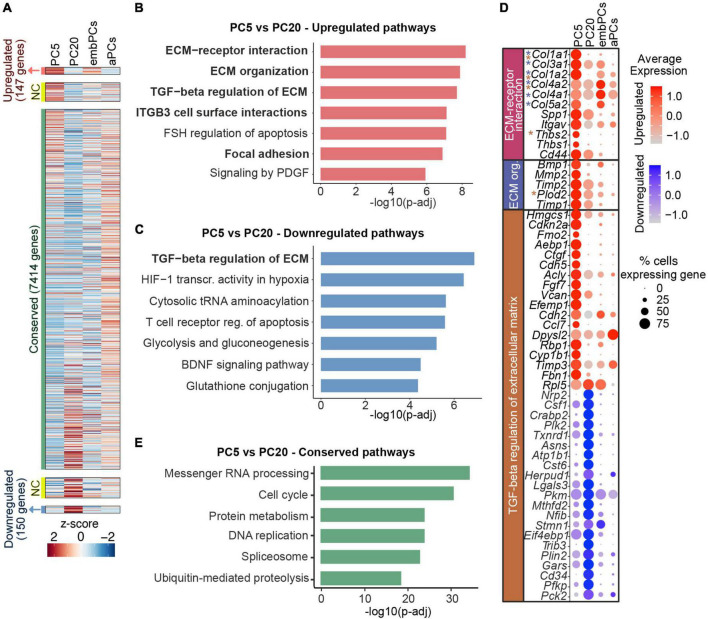
Long-term PC culture results in reduction of collagen expression. **(A)** Heatmap representing enriched expression of genes in PC5 and PC20. Colors represent the average z-score of a cluster for each gene. NC indicates not considered genes (0.5 < log_2_FC < 1 or–1 < log_2_FC < –0.5). **(B)** BioPlanet annotated pathways significantly upregulated in PC5 vs. PC20. **(C)** BioPlanet annotated pathways significantly downregulated in PC5 vs. PC20. **(D)** Dot plot representing the average expression of deregulated genes belonging to up/downregulated pathways in PC5. Color scales indicate if the gene is upregulated (red) or downregulated (blue) in PC5 vs. PC20. Genes with asterisks are also part of other deregulated pathways (i.e., blue for the ECM organization pathway, and brown for the TGF-beta regulation of extracellular matrix). **(E)** BioPlanet annotated pathways conserved in PC5 vs. PC20. aPCs, adult brain pericytes; ECM, extracellular matrix; embPCs, embryonic brain pericytes; NC, not considered; org., organization; PCs, pericytes; PC5, passage 5 pericytes; PC20, passage 20 pericytes; TGF, transforming growth factor.

Despite being very similar at the transcriptional level (*r* = 0.89, Figure 2A), we next addressed how cultured PCs change from passage 5 to 20. Out of the 8523 genes expressed in PC5 and PC20, 147 were upregulated (log_2_FC > 1), 150 were downregulated (log_2_FC < −1) and 7414 were conserved (−0.5 < log_2_FC < 0.5) in PC5 (Figure 4A). Interestingly, the analysis showed a higher expression of pathways associated with ECM interaction and organization in PC5 (Figure 4B). Additionally, some genes related to the TGF-β regulation of ECM were deregulated in PC5 (Figures 4B, C). This indicates that prolonged culturing of primary mouse brain PCs leads to the decline and deregulation of important ECM factors, in particular collagens (*Col1a1*, *Col1a2*, *Col3a1*, *Col4a1*, *Col4a2*, *Col5a2*), which are downregulated at passage 20 (Figure 4D). Passage 5 PCs also displayed a downregulation of genes in the glycolysis and gluconeogenesis pathways when compared to PC20 (Figures 4C, D). These results demonstrate a metabolic shift in cells at a later passage. However, housekeeping pathways were highly conserved in cultured PCs, for example, messenger RNA processing, cell cycle and protein metabolism (Figure 4E). Taken together, these data suggest that even though passage 5 and 20 PCs have very similar transcriptional profiles, they differ in the expression of ECM-related and glycolytic genes.

### 3.5. Expression of canonical PC markers and ECM-related genes is improved by co-culture with brain ECs

**FIGURE 5:**
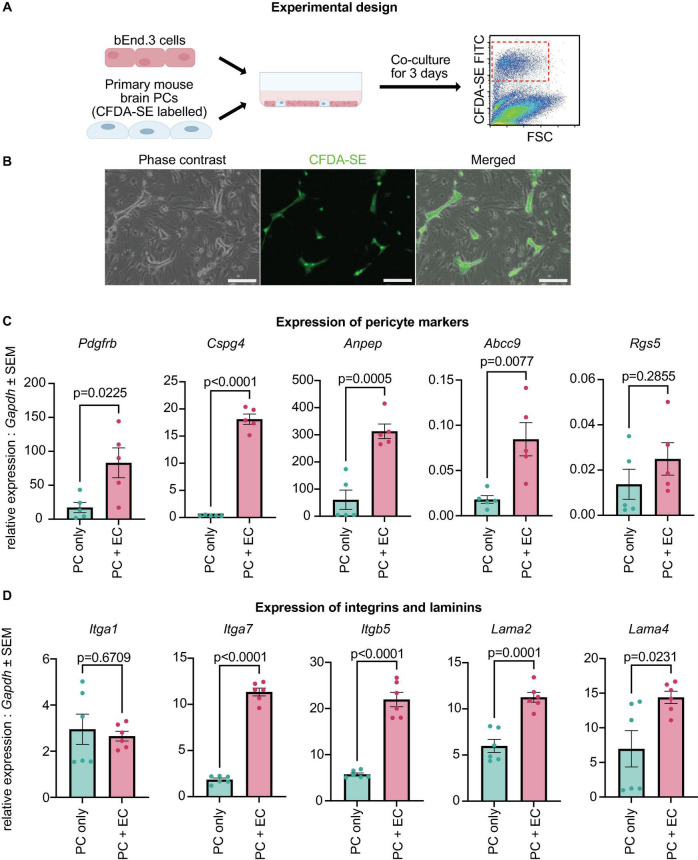
Canonical PC markers and ECM genes are upregulated with brain EC co-culture. **(A)** Experimental design: primary brain PCs were labeled, co-cultured with bEnd.3 cells for 3 days, isolated by FACS sorting, and analyzed via qRT-PCR. **(B)** Localization of stained PCs within the co-culture with bEnd.3 cells. Scale bar, 200 μm. **(C)** Expression levels of *Pdgfrb*, *Cspg4*, *Anpep*, *Abcc9*, and *Rgs5* in PCs cultured alone (PC only) and co-cultured with bEnd.3 cells (PC + EC). The data were normalized to *Gapdh* and analyzed with a two-sided Student’s *t*-test. Data are presented as mean ± SEM. The experiment was repeated 5 times, with each data point indicating a replicate. **(D)** Expression levels of *Itga1*, *Itga7*, *Itgb5*, *Lama2*, and *Lama4* in PCs cultured alone (PC only) and co-cultured with bEnd.3 cells (PC + EC). The data were normalized to *Gapdh* and analyzed with a two-sided Student’s *t*-test. Data are presented as mean ± SEM. The experiment was repeated 6 times, with each data point indicating a replicate.

In our data, cultured PCs displayed low expression of canonical PC markers (Figure 1G). As PDGF-B secreted by endothelial cells (ECs) is essential for the establishment of PCs in the microvasculature (Bjarnegaård et al., 2004), we hypothesized that the downregulation of canonical PC markers in cultured PCs might be due to the lack of interaction with ECs. Therefore, we cultured early passage brain PCs (passage 8) with bEnd.3 brain endothelial cells for 3 days and quantified the expression of canonical PC markers (Figures 5A, B). Passage 8 primary brain PCs that had not been cultured with brain ECs were used as controls. Interestingly, PCs co-cultured with bEnd.3 ECs had a significantly higher expression of *Pdgfrb*, *Cspg4*, *Anpep*, and *Abcc9* when compared to PCs in a monoculture, while *Rgs5* expression was unchanged between the two groups (Figure 5C). This result indicates that PCs require interactions with ECs in order to properly express their canonical markers.

Our single cell analysis also uncovered the downregulation of ECM-related genes in cultured PCs when compared with both embryonic and adult brain PCs (Figures 2G, 3F). In particular, integrins (*Itga1*, *Itga4*, *Itga7*, *Itgb5*) and laminins (*Lama2*, *Lama4*, *Lamb2*, *Lamc3*) displayed a significantly lower expression in PC5 and PC20, suggesting that the extracellular matrix might be disrupted *in vitro* (Nirwane and Yao, 2019; Halder et al., 2022). We tested if laminins and integrins were upregulated in our co-culture model. The expression of *Itga7*, *Itgb5*, *Lama2*, and *Lama4*, but not *Itga1*, was significantly higher in PCs co-cultured with ECs (Figure 5D). Therefore, the interaction between the endothelium and PCs is important for the expression of ECM-related genes.

## 4. Discussion

In this study, we provide evidence that cultured PCs are a highly homogenous population of cells that share a lot of similarities with embryonic brain PCs. Housekeeping gene expression is conserved across cultured, adult and embryonic PCs. However, both early and late passage PCs display low expression of canonical PC markers and show downregulation of ECM genes, in particular integrins and laminins. These expression of these genes was increased by the co-culture of PCs with ECs, indicating that the microvascular environment is important to maintain PC identity.

Pericytes are critical for maintaining brain function, by regulating vascular integrity, immune responses and vascular transport (Bell et al., 2010; Armulik et al., 2011). Thus, the culture and mechanistic analysis of PCs has become a central tool to better understand the molecular networks that regulate their functions. The culture of PCs typically requires extraction of vascular cells or vascular fragments, followed by serial passaging to obtain a pure PC monoculture (Balabanov et al., 1996; Boroujerdi et al., 2014). Selective propagation of primary PCs can be performed with either DMEM supplemented with F12, serum and antibiotics (Nakagawa et al., 2009; Nees et al., 2012; Thomsen et al., 2015; Smyth et al., 2018b; Stevenson et al., 2022), or with a commercially available PC medium as we have done in this study (Tigges et al., 2012; Boroujerdi et al., 2014). PCs cultured in the two different media show differences in proliferation rates, while PCs cultured in commercial media show lower expression of canonical PC markers relative to the DMEM/F12 medium (Rustenhoven et al., 2018). Nevertheless, PCs in both media display comparable responses to cytokines such as IL1β, to growth factors such as PDGF-BB, and similar phagocytic uptake (Rustenhoven et al., 2018). This suggests that the results obtained from the two different media are comparable. Given the similarities in the establishment and propagation of PCs across different studies, and the response of PCs to environmental stimuli regardless of media, our analysis here are likely to broadly applicable to PC cultures used in other studies.

Our initial analysis indicated that well-established PCs markers were downregulated *in vitro*. Consistent with our findings, lower transcriptional levels of PC markers have also been observed in cultured human cortex PCs (Gastfriend et al., 2021). However, other authors have shown that the proteins of PC markers can be detected in cultured human brain PCs (Smyth et al., 2018b) and mouse brain PCs (Tigges et al., 2012; Hutter-Schmid and Humpel, 2018). Since the interaction with ECs is essential for PC recruitment and differentiation (Enge et al., 2002; Bjarnegaård et al., 2004), we tested if the co-culture of PCs and ECs could recover the levels of selected PC markers. Our data shows that *Pdgfrb*, *Cspg4*, *Anpep*, and *Abcc9* were significantly upregulated in co-cultured PCs. Similar to our results, the co-culture of human ECs with PCs has been described as essential for the retention of maturity markers and the stimulation of PC proliferation *in vitro* (Brandt et al., 2019). Therefore, the state of PCs should be carefully considered when performing an experiment with cultured PCs, since PCs require ECs to maintain their maturity and identity.

The DE analysis of our data indicated a strong downregulation of multiple laminins (*Lama2*, *Lama4*, *Lamb2*, *Lamc3*) and integrins (*Itga1*, *Itga4*, *Itga7*, *Itgb5*) in cultured PCs. Laminins are an important constituent of the basement membrane (BM), which is a unique form of highly organized ECM (LeBleu et al., 2007; Nirwane and Yao, 2019). Cells are anchored to the surrounding ECM by plasma membrane proteins known as integrins (Kechagia et al., 2019). In the brain microvasculature, laminin synthesis and deposition to the BM is collectively done by ECs, PCs and astrocytes (Nirwane and Yao, 2019; Halder et al., 2022). Therefore, we tested if the expression of laminins and integrins could be upregulated in PCs cultured with ECs. In our data, the expression of *Lama2*, *Lama4*, *Itga7*, and *Itgb5* was higher in PCs sorted from a co-culture with ECs (Figure 5D). Similar to our results, it has been shown that laminin-α4, α5, β2, and γ1 deposition to the BM *in vitro* is greatly increased when ECs and PCs are in a co-culture (Stratman et al., 2009). These data suggested that the contact with ECs is essential for the proper expression of ECM genes in PCs. This likely reflects the notion that PCs require ECs for their maturation.

Sex is an important regulator of cellular phenotypes and the transcriptome (Oliva et al., 2020). For instance, 14 to 25% of the endothelial transcriptome is thought to be influenced by sex (Hartman et al., 2020). Our study primarily focused on cells extracted from female animals (Supplementary Figure 1G). Indeed, cultured PCs, aPCs, SMCs and ECs were all extracted solely from females. The embPC population contained a mix of male and female cells (Supplementary Figure 1G). However, the cells from embPCs clustered together regardless of sex, suggesting that sex did not have an overall impact on the transcriptional profile of embPCs. Given that our analyses were primarily extracted solely from female mice, they are likely to represent biological rather than sex differences. Nevertheless, future studies will be required to ascertain whether PCs extracted from male animals show the same behavior as documented here for females.

In this study, we have utilized state-of-the-art single cell technologies to show that cultured PCs are a homogenous population that are more similar to embryonic PCs. Indeed, both embPCs and cultured PCs express cell proliferation genes, while low expression of canonical PC markers and deregulation of ECM genes are evident in cultured PCs. However, the presence of brain ECs in co-culture conditions can promote a more mature state of PCs, with an upregulation of canonical PC markers and ECM components. It is important to consider these key differences prior to utilizing primary brain PCs as an *in vitro* model to study molecular and functional changes in neural homeostasis and disease.

## Data availability statement

The datasets presented in this study can be found in online repositories. The names of the repository/repositories and accession number (s) can be found below: https://www.ncbi.nlm.nih.gov/, GSE98816; https://www.ncbi.nlm.nih.gov/, GSE133079; and https://www.ncbi.nlm.nih.gov/, GSE225219.

## Ethics statement

The animal study was reviewed and approved by the Landesdirektion Sachsen, Leipzig.

## Author contributions

FO, OB, and BS conceptualized, initiated, and developed the project. OB processed pericytes for single cell analysis. FO undertook the bioinformatics data analysis. FO and JR-A carried out downstream experiments. OB and BS supervised the project. FO and BS wrote the manuscript. All authors read, edited, and approved the manuscript.

## Supplementary material

Supplementary Figure 1 Data processing and cluster identification. **(A)** Phase contrast pictures of passage 9 and passage 22 PCs. Scale bar equals 200 μm. **(B)** Violin Plot representing the number of features (i.e., number of unique genes identified per cell), number of counts per cell, percentage of mitochondrial genes and percentage of ribosomal genes in the datasets included in this study. The number of counts are shown on a log scale. The pericyte P5, P20, and brain_pericyte datasets were generated in this study. The brain Pdgfra, mural and EC data were derived from Vanlandewijck et al. (2018). The embryonic pericyte data were derived from Sheikh et al. (2019). **(C)** Preliminary UMAP clustering of all cells included in the study. Colors and numbers indicate the total 18 clusters identified in the analysis. **(D)** Correlation of gene expression across the initial 18 clusters. Pearson’s *r*-values were computed and are represented by the red color. **(E)** UMAP representing the original dataset of each cell. **(F)** Heatmap indicating the 50 most enriched genes in each cluster. The color scale represents the average z-score for a specific gene across the clusters. **(G)** UMAP showing the expression of the sexually dimorphic genes *Xist* and *Ddx3y*. **(H)** Enrichment of established cell markers for pericytes (PCs), smooth muscle cells (SMC), endothelial cells (ECs), fibroblasts, oligodendrocytes (OL), microglia, astrocytes and mesenchymal stem cells (MSC) across the 10 major clusters (Han et al., 2018; He et al., 2018; Vanlandewijck et al., 2018). The heatmap represents the z-score for each cluster.

Supplementary Figure 2 Comparison of cultured and adult PCs. **(A)** Enrichment of all genes expressed in PC5 and aPCs. Heatmap depicts the average z-score of a cluster for each gene. NC indicates not considered genes with either 0.5 < log_2_FC < 1 or −0.5 > log_2_FC > −1. **(B)** Enrichment of all genes expressed in PC20 and aPCs. Heatmap represents the average z-score of a cluster for a specific gene. NC indicates not considered genes (0.5 < log_2_FC < 1 or −0.5 > log_2_FC > −1). **(C)** BioPlanet annotated pathways significantly upregulated in PC5 in comparison to aPCs. **(D)** BioPlanet annotated pathways significantly downregulated in PC5 in comparison to aPCs. **(E)** BioPlanet annotated pathways significantly upregulated in PC20 in comparison to aPCs. **(F)** BioPlanet annotated pathways significantly downregulated in PC20 in comparison to aPCs. aPCs, adult brain pericytes; embPCs, embryonic brain pericytes; PCs, pericytes; PC5, passage 5 pericytes; PC20, passage 20 pericytes.

Supplementary Table 1 List of primers used in the study and their sequences.
