# Supplementary figures and images for: Cultured brain pericytes adopt an immature phenotype and require endothelial cells for expression of canonical markers and ECM genes
Source: Front Cell Neurosci. 2023 May 2;17:1165887. doi: 10.3389/fncel.2023.1165887 (PMC10185779; doi:10.3389/fncel.2023.1165887)

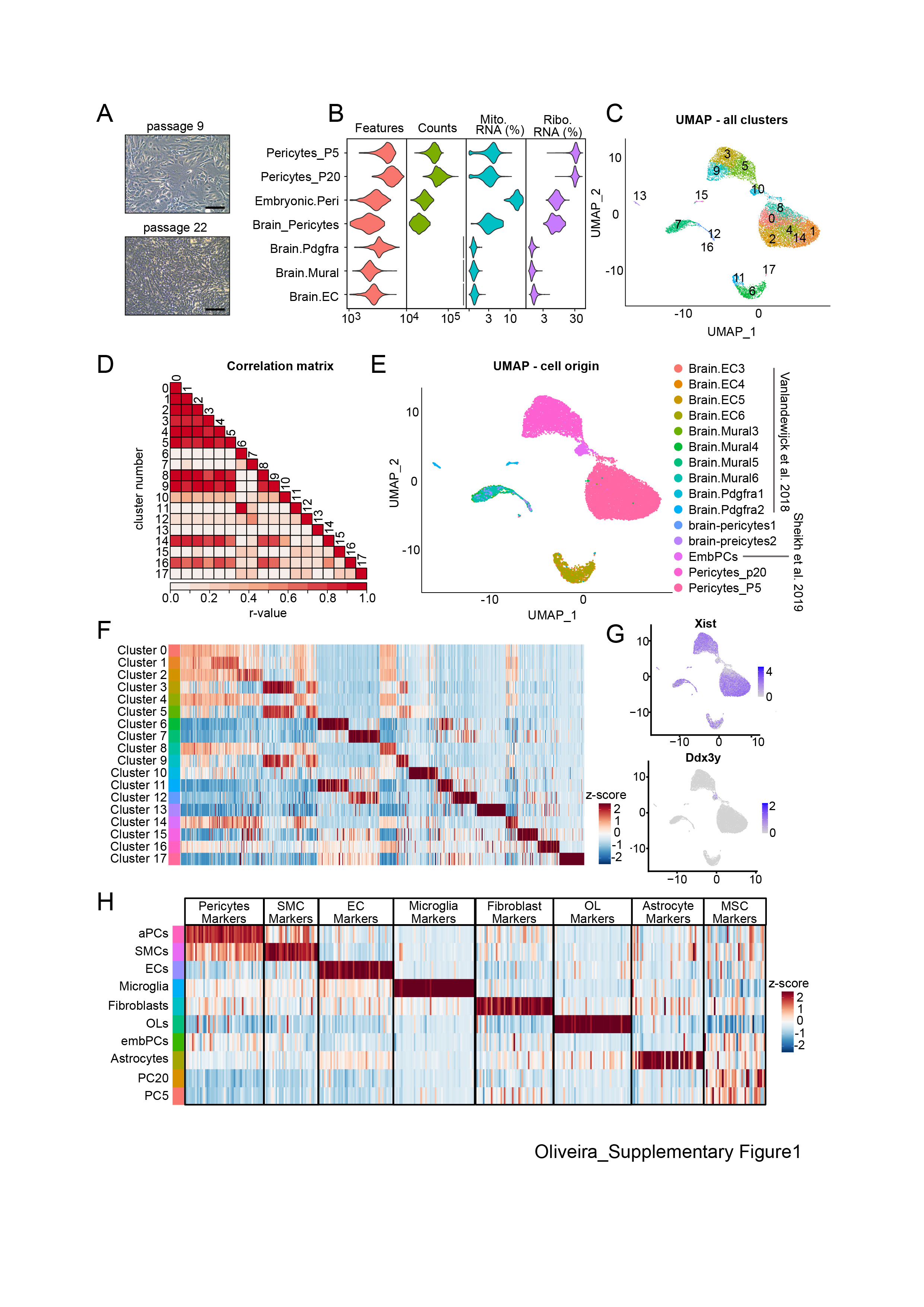

Supplement: Supplementary Figure 1 — Data processing and cluster identification. (A) Phase contrast pictures of passage 9 and passage 22 PCs. Scale bar equals 200 μm. (B) Violin Plot representing the number of features (i.e., number of unique genes identified per cell), number of counts per cell, percentage of mitochondrial genes and percentage of ribosomal genes in the datasets included in this study. The number of counts are shown on a log scale. The pericyte P5, P20, and brain_pericyte datasets were generated in this study. The brain Pdgfra, mural and EC data were derived from Vanlandewijck et al. (2018). The embryonic pericyte data were derived from Sheikh et al. (2019). (C) Preliminary UMAP clustering of all cells included in the study. Colors and numbers indicate the total 18 clusters identified in the analysis. (D) Correlation of gene expression across the initial 18 clusters. Pearson’s r-values were computed and are represented by the red color. (E) UMAP representing the original dataset of each cell. (F) Heatmap indicating the 50 most enriched genes in each cluster. The color scale represents the average z-score for a specific gene across the clusters. (G) UMAP showing the expression of the sexually dimorphic genes Xist and Ddx3y. (H) Enrichment of established cell markers for pericytes (PCs), smooth muscle cells (SMC), endothelial cells (ECs), fibroblasts, oligodendrocytes (OL), microglia, astrocytes and mesenchymal stem cells (MSC) across the 10 major clusters (Han et al., 2018; He et al., 2018; Vanlandewijck et al., 2018). The heatmap represents the z-score for each cluster. [file Image_1.JPEG]

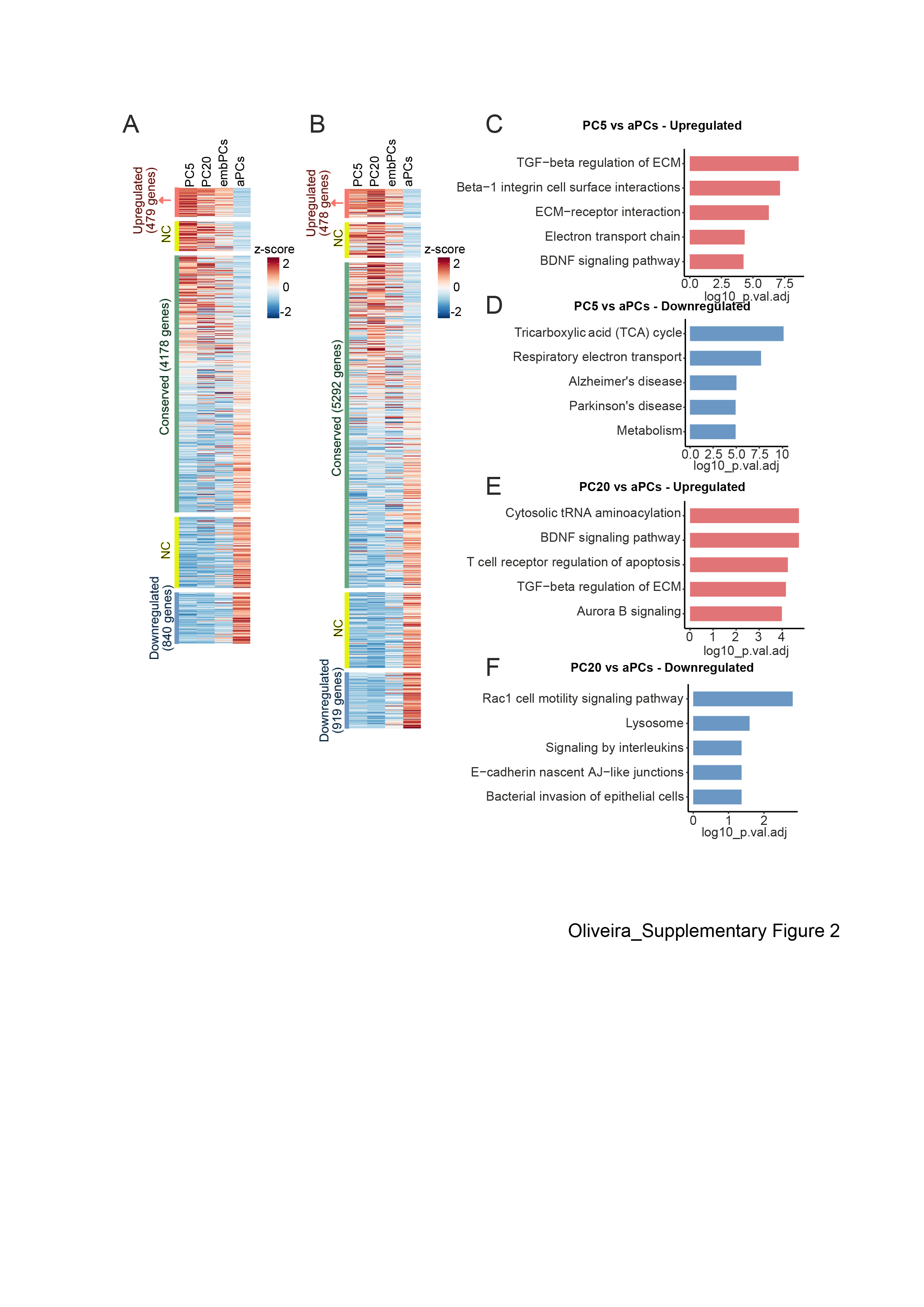

Supplement: Supplementary Figure 2 — Comparison of cultured and adult PCs. (A) Enrichment of all genes expressed in PC5 and aPCs. Heatmap depicts the average z-score of a cluster for each gene. NC indicates not considered genes with either 0.5 < log2FC < 1 or −0.5 > log2FC > −1. (B) Enrichment of all genes expressed in PC20 and aPCs. Heatmap represents the average z-score of a cluster for a specific gene. NC indicates not considered genes (0.5 < log2FC < 1 or −0.5 > log2FC > −1). (C) BioPlanet annotated pathways significantly upregulated in PC5 in comparison to aPCs. (D) BioPlanet annotated pathways significantly downregulated in PC5 in comparison to aPCs. (E) BioPlanet annotated pathways significantly upregulated in PC20 in comparison to aPCs. (F) BioPlanet annotated pathways significantly downregulated in PC20 in comparison to aPCs. aPCs, adult brain pericytes; embPCs, embryonic brain pericytes; PCs, pericytes; PC5, passage 5 pericytes; PC20, passage 20 pericytes. [file Image_2.JPEG]
